# Applying the syndemic framework to cancer research for effective cancer control in low- and middle-income countries

**DOI:** 10.3332/ecancer.2023.1532

**Published:** 2023-04-20

**Authors:** Mariam Hassan, Zahid Ahmad Butt

**Affiliations:** 1 Shaukat Khanum Memorial Cancer Hospital and Research Centre, Lahore 54000, Pakistan; 2 School of Public Health Sciences, University of Waterloo, Waterloo, ON N2L 3G1, Canada

**Keywords:** cancer, low- and middle-income countries, social determinants of health, disease clustering, syndemics, social context, health outcomes

## Abstract

Cancer burden is increasing rapidly globally, especially in low- and middle-income countries (LMICs), which already face a double burden of infectious diseases and other non-communicable diseases (NCDs). LMICs also struggle with poor social determinants of health, leading to cancer health disparities, such as delayed diagnoses and increased death rates due to cancer. Contextually, relevant research needs to be prioritised in these regions to ensure feasible, evidence-based healthcare planning and delivery for cancer prevention and control. A syndemic framework has been used to study the disease clustering of infectious diseases and NCDs across varied social contexts to understand how diseases interact adversely and how the wider environmental context and other socioeconomic factors contribute to poor health outcomes within specific populations. We propose using this model to study the ‘syndemic of cancers’ in the disadvantaged population of LMICs and suggest ways for the clear operationalisation of the syndemic framework through multidisciplinary evidence-generation models for the delivery of integrated, socially conscious interventions for effective cancer control.

## Background

The global cancer burden is expected to rise substantially in the next 50 years, with a 400% increase seen in low- and middle-income countries (LMICs) due to population expansion, improved life expectancy, urbanisation and lifestyle changes leading to a rise in risk factors, such as obesity and exposure to tobacco [1]. Inequalities in social determinants of health, including gender inequality in LMICs, also contribute to persistent disparities, such as higher cancer deaths in these populations. It is expected that, by the year 2030, approximately 70% of cancer deaths occur in LMICs [2]. The rising burden of cancer in LMICs stresses an already weak health care and economic infrastructure leading to poorer outcomes. Cancer research to generate country-specific evidence has not been recognised as a high-priority area in these countries, despite these facts, and ongoing efforts focus predominantly on expanding and strengthening treatment facilities. It is imperative that LMICs must conduct their own research relevant to local needs, which yields feasible, effective and implementable solutions locally and could also impact cancer control globally [3].

LMICs also face a double burden of infectious diseases that continue to pose threats alongside rising morbidity and mortality from non-communicable diseases (NCDs), including cancer. This poses a complex medical context in which NCDs interact with chronic infectious diseases present in these populations. A growing body of research shows the negative effect that NCD-infectious disease convergence has, especially on low-income groups within LMICs. However, global health priorities and financing still largely focus on infectious diseases, consequently leading to overburdening health systems with people seeking care in the advanced stages of NCDs [4].

## Syndemic framework

Understanding how certain diseases cluster across varied social contexts and within specific populations is necessary and can be studied using the syndemic framework as a tool. A syndemic involves two or more diseases that interact to worsen health outcomes and includes consideration of how the wider environmental context and other socio-economic and political factors contribute over time to mutually exacerbate negative outcomes [5]. The term ‘syndemic’ was first used by Singer [6], a medical anthropologist who explored how substance abuse, violence and AIDS (acquired immunodeficiency syndrome) (SAVA) cluster together and affect one another in a disadvantaged community in the USA. A range of syndemic studies have been reported in the last 30 years, illustrating the framework’s applicability to other public health conditions. Syndemics are not limited to infectious diseases. The VIDDA syndemic describes how violence, immigrant status and isolation, depression, type 2 diabetes and abuse interact adversely and exacerbate negative health outcomes in a group of immigrant women in the USA and is an example of how NCDs and health conditions cluster and interact [7]. A World Health Organization health survey of chronic diseases (angina, arthritis, asthma and diabetes) in the adult population from 60 countries showed that co-occurring depression worsened health outcomes beyond what was found with any of the chronic diseases or with any combination of the four chronic diseases without depression. In addition, there is increasing evidence that major depression is much more common in those with chronic conditions like cardiovascular disease, diabetes and cancer than in the general population, especially among people living in poverty [4]. The COVID-19 pandemic has also demonstrated how pre-existing NCDs, such as cancer when situated within contexts of poor social and political determinants of health, have a profound effect on COVID-19 vulnerability and its outcome, hence understanding the biosocial context is the key to effective public health programmes [8, 9].

## Applying the syndemic framework for cancer control

Syndemics, in short, occur across the disease spectrum and often involve adverse interactions among varied biological and social conditions. The theory draws attention to and provides a specific framework of adverse biological interaction between all types of diseases (e.g., infections, chronic NCDs, mental health and environmental exposure-related illnesses, and malnutrition) and enables analysis of the impact of social conditions (health inequity caused by poverty, violence, stress, stigmatisation and gender inequality) to identify factors that may contribute to disease clustering, spread, increased susceptibility and progression. By understanding how disease interactions may lead to adverse health outcomes, we can evaluate how social and economic contexts exacerbate disease clusters and what health interventions might be most effective for these conditions [10, 11]. A study of the geographical distribution of county-level prevalence of major chronic conditions and mortality rates among the socially vulnerable counties in the USA showed that social disadvantage is strongly linked to factors that exacerbate infectious disease epidemics and that such populations were also more likely to suffer from chronic conditions that worsened their health outcomes. However, it is also noteworthy that some counties had a lower prevalence of chronic conditions, despite higher social vulnerability highlighting the role of health and social policies (e.g., an increase in the cigarette tax to address smoking) in these states [12]. This highlights that public health and social policy measures are central to the control of the syndemic by identifying the social, economic, and geographic contexts that co-interact and cumulatively impact outcomes. This approach could help address the multiple risk factors for cancer control and identify disparities around cancer diagnosis and treatment.

Syndemic theory also highlights the need to identify best practices for the simultaneous treatment of interlocked conditions. NCDs share risk factors resulting in the escalation of comorbidities, especially among marginalised populations; hence, there is a clear need for integrated care that addresses the structural and social problems affecting chronic diseases. Although this may appear outside the scope of clinical medicine, there is precedent for such syndemic care models that treat multiple disorders and specific contextual vulnerabilities holistically, such as Rwanda’s Ministry of Health with Partners in Health and the Clinton Foundation, who, working together, were able to design NCD care for those living with AIDS in a largely rural and decentralised healthcare delivery system. This model focused on aspects that make HIV (Human Immunodeficiency Virus) a social disease, such as poverty and racism. Other integrative programmes, such as Academic Model Providing Access to Healthcare, also show that syndemic care available at the primary care level, as well as the community level through integrated healthcare and the strengthening of health systems, improves health outcomes [4].

Using similar syndemic care models for cancer control would be especially useful for limited resource settings as they can help ensure improvement in both individual and wider public health outcomes as multiple factors and their shared context is being considered for delivering an intervention [13]. The current models for oncologic care in most LMICs focus on diagnosis and treatment without ways to address structural and social vulnerabilities that affect outcomes. A syndemic framework can be adopted as a tool for the assessment and delivery of integrated health programmes, especially those dealing with multiple chronic conditions. For example, tobacco use has been shown to cluster with the rise of NCDs, such as cancer, cardiovascular disease and diabetes, in Pacific Islanders, which cannot be separated from the introduction of industrially manufactured cigarettes to Pacific Islanders by tobacco companies. In this population, the cluster of NCDs among smokers has emerged when factors such as corporate exploitation, poverty and poor healthcare access worsen the biological interactions among these conditions and their outcomes [14]. Models to support the implementation of a syndemic approach for the prevention and control of diseases have been reported in September 2010 in six health departments across New York City, North Carolina, Philadelphia, San Francisco, Texas, and Washington, DC. They were funded with $6.2 million by the Centres for Disease Control and Prevention, USA, for Program Collaboration and Service Integration (PCSI) demonstration projects to plan, scale up and support the implementation of a syndemic approach for the prevention of HIV/AIDS, viral hepatitis, sexually transmitted diseases and tuberculosis. PCSI is a strategy intended to strengthen collaborative work across programmes for efficiency and integration of services to the public. The goal was to collaboratively develop a sustainable system of primary prevention and clinical care to prevent transmission, disease, disability and death; to reduce co-infections; and to increase health equity. Surveillance baseline assessments in these programmes enabled direct assessment of the extent of patient-based overlap between diseases of interest using registry matches. Visualising the syndemic to identify populations at risk for multiple diseases provided critical input for integrating preventive services at the point of care. This was followed by identifying a baseline for integrated services and gathering stakeholder input from health care staff as well as community planning groups and then using that data to develop plans for the integrated delivery of new screening recommendations and for measuring the impact of the new recommendations on the level of integrated services. The demonstration project accomplishments included integrated screening and health services delivery, integrated IT (information technology) solutions and data sharing through collaborations and public health reorganisation models. PCSI appeared as an important tool that can help give programmes enhanced flexibility, efficiency and control in the delivery of services in the face of evolving epidemiology, common risk behaviours, similar modes of transmission and concurrent disease interactions [15].

Similar frameworks can be used for cancer; however, this requires a clear operationalisation of the syndemic framework and a clear study of how social and medical problems cluster and interact within certain populations. A review of the syndemic research highlights that studies of NCD-related syndemics tended to focus on micro-level context, pointing towards methodological gaps (such as a lack of mixed methods studies) and the challenges associated with interdisciplinary research. The meta-knowledge study of existing syndemic literature also highlights the need for studying this framework for the population in LMICs to inform evidence-based health policy-making that is inclusive and context-appropriate to advance global health for the most disadvantaged populations [16, 17]. Effective research on syndemic problems thus requires taking methodological approaches that allow for the simultaneous exploration of diseases and their social contexts. One such methodological approach is complex systems analysis, which involves understanding a system consisting of elements (sociological context), relationships that hold elements together, such as policies/laws, individual-level risk-reduction practices (interconnections) and the functions of the system. These components make up the dynamics of a social context that drives a syndemic, and hence studying these can be useful. In addition, ethnography, a descriptive and inductive research method, can provide rich insights into factors that interact to shape social contexts that may increase the risk of a syndemic in a particular population. This approach was not only used in describing the SAVA syndemic but also in recent works on exploring the heightened risk of sexually transmitted infections in marginalised youth. Furthermore, large-scale longitudinal studies (employing advanced analytical models) that look at both disease patterns in individuals as well as the social condition and public health research that integrates the record of prevention and treatment of diseases, as well as the factors that contribute to risk and vulnerability in the studied population, can be highly valuable for syndemic research [18].

Hence, evidence for the detection of syndemics and effective integrated care delivery needs multidisciplinary evidence generation models with robust empirical analytic approaches including (but not limited to) social and spatial epidemiology, quantitative research supported by ethnography as well as clinical trials to determine if the syndemic approach is effective for cancer control [17, 19].

Studying syndemics using these multidisciplinary models would require the formation of research teams with varied areas of expertise, including the fields of public health, medicine, nursing, epidemiology, anthropology, sociology, mental health, computer science, statistics, and behavioural sciences, as well as community groups and experts. This would allow for a diverse stakeholder collaboration, which would not only enable the examination of factors spanning disciplines and multiple levels of analysis but also carefully consider how cultural forces act upon both the communities impacted by a syndemic and the course of the research itself. In addition, ethnically and racially diverse research workforce with experience in working and living within communities affected by syndemics may allow for an enhanced understanding of the functioning of a syndemic within a particular community as well as implementing programs and policies that can effectively control and ultimately eradicate a syndemic [18, 20].

Innovation and community-driven solutions, which are based on local epidemiology and social determinants of health data, are at the heart of programmes that have attempted to take a syndemic approach to control of disease in specific populations [21]. This necessitates expanding the availability of funding to grassroots and community-based organisations to address systemic challenges such as housing insecurity and mental health care that often impact access to preventive health. Similarly, studying social determinants of health, including gender (and its related concepts such as masculinity, patriarchy and heteronormativity) and their impact on health outcomes through differentiated risk exposures, use of and access to healthcare services, and gender bias in health systems can help operationalise such social phenomena that impact health equity [22]. This can only be achieved if funding bodies reserve an appropriate amount of collaborative research grants specifically to support work that adopts a syndemic approach. Funders should also demand rigorous evidence for investments in interventions leading to improved health outcomes and to maximise efficiencies based on the most cost-effective, highest impact and feasible approaches for the most vulnerable populations.

## Conclusion

Cancer is a complex disease, and its rising burden in LMICs requires the integration of services that extend beyond healthcare delivery, for effective cancer control. This requires prioritising research that looks at risk and prognostic factors for cancer and other NCDs, especially among low-income, marginalised populations. The clustering of social and health problems underlies the delayed diagnosis, poor access and utilisation of cancer control services as well as poor cancer survivorship in those living with and beyond cancer, ultimately leading to adverse outcomes in these populations.

Syndemic research can help operationalise how social problems cluster with and affect medical problems and help design interventions that address these structural inequities for effective cancer control. Although this issue may appear to be outside the scope of expertise of those providing clinical care, we argue that understanding how structural and contextual factors drive fragmented care for cancer is essential for planning better cancer care and delivery.

## Conflicts of interest

The authors declare that they have no known competing financial interests or personal relationships that could have appeared to influence the work reported in this paper.

## Funding

This work did not receive any specific grant from funding agencies in the public, commercial, or not-for-profit sectors.

## Author contributions

Mariam Hassan – Conceptualization, Methodology, Writing–Original draft preparation, Writing–Reviewing and Editing. Zahid Butt – Conceptualization, Supervision, Writing–Reviewing and Editing.
